# Supplementary material for: Complexome profiling of the Chlamydomonas psb28 mutant reveals TEF5 as an early PSII assembly factor
Source: Plant Cell. 2025 Mar 17;37(6):koaf055. doi: 10.1093/plcell/koaf055 (PMC12164590; doi:10.1093/plcell/koaf055)
Supplement: koaf055_Supplementary_Data [file koaf055_supplementary_data.zip › psb28_interactive.zip!/plots/intensity/qualityPlots/peptides/_Cre12.g494350.t1.2.html]

### Cre12.g494350.t1.2

Trivial name:   
SeqCoverage [median]: 8.50  
Mass: 76.09 kDa  
  
 

| Sequence | MissedCleavage | Mass [Da] | wtIntensity | muIntensity | Score |
| --- | --- | --- | --- | --- | --- |
| DLQKYEALVVEGGGGPDSR | 1 | 1988.98 | 2401.35 | 5615.09 | 92.39 |
| IANTANPGTILEGIR | 0 | 1538.84 | 1942.13 | 12467.38 | 184.11 |
| LVAGDVFRPPANSASLAVR | 1 | 1939.06 | 5511.98 | 10461.14 | 107.75 |
| TNQIPR | 0 | 727.40 | 3769.25 | 5958.67 | 140.01 |
| YEALVVEGGGGPDSR | 0 | 1504.72 | 298.36 | 823.14 | 178.32 |
|  |  |  | 13923.1 | 35325.4 |  |
